# Age-specific performance of human papillomavirus E6/E7 mRNA assay versus cytology for primary cervical cancer screening and triage: community-based screening in China

**DOI:** 10.3389/fcimb.2024.1428071

**Published:** 2024-08-29

**Authors:** Jing Zhang, Guangcong Liu, Di Yang, Xiaoli Cui, Chunyan Wang, Danbo Wang, Haozhe Piao

**Affiliations:** ^1^ Department of Gynecology, Cancer Hospital of China Medical University, Liaoning Cancer Hospital and Institute, Shenyang, Liaoning, China; ^2^ Department of Epidemiology, Cancer Hospital of China Medical University, Liaoning Cancer Hospital and Institute, Shenyang, Liaoning, China; ^3^ Department of Neurosurgery, Cancer Hospital of China Medical University, Liaoning Cancer Hospital and Institute, Shenyang, Liaoning, China

**Keywords:** age group, cervical screening, cytology, HPV E6/E7 mRNA, triage

## Abstract

**Background:**

In the general population, primary human papillomavirus (HPV) testing is advocated for cervical cancer (CC) screening. HPV E6/E7 mRNA (Aptima HPV, AHPV) assays have garnered considerable traction due to their higher specificity when compared with HPV DNA assays. Here, we investigated age-specific primary AHPV screening assays and different triage strategies versus cytology to identify the best approach.

**Methods:**

Between April 2018 and December 2021, we recruited female participants from 34 communities across Liaoning province and Qingdao City, China. Primary cervical screening protocols included liquid-based cytology (LBC) and AHPV assays, with females positive for any assays undergoing colposcopy. Genotyping (AHPV-GT) was conducted on all HPV-positive samples. Our primary outcomes were the identification of age-specific detection rates, colposcopy referral rates, and sensitivity and specificity values for high-grade squamous intraepithelial lesions or worse (HSIL+). AHPV and different triage strategy performances were also examined across different age cohorts.

**Results:**

Our investigation included 9911 eligible females. Age-specific abnormal cytology rates were in the 6.1%–8.0% range, and were highest in 45–54-year olds. When compared with 35–44-or 45–54-year olds, HPV prevalence was highest in 55–64-year olds (12.2% or 11.6% vs.14.1%, P = 0.048 and P = 0.002, respectively). In 35–44-year olds, AHPV sensitivity for detecting HSIL+ was 96.6 (95% confidence interval [CI]: 89.7–100) - significantly higher than LBC sensitivity (65.5 [95% CI: 48.3–82.8], P < 0.001). When compared with LBC, HSIL+ detection rates by AHPV-GT using reflex LBC triage increased by 31.5% (9.6‰ vs. 7.3‰), and colposcopy referral rates decreased by 16.4% (5.1% vs. 6.1%). In 45–54-year olds, HSIL+ detection rates for AHPV-GT using reflex LBC triage were lower than LBC rates (6.2‰ vs. 6.6‰). In 55–64-year olds, AHPV sensitivity (97.2 [95% CI: 91.7–100.0]) was higher than LBC sensitivity (66.7 [95% CI: 50.0–80.6], P = 0.003). The area under the curve (AUC) value was not significantly different between AHPV-GT with reflex LBC triage and LBC (0.845 [95% CI: 0.771–0.920] vs. 0.812 [95% CI: 0.734–0.891], P = 0.236).

**Conclusions:**

Primary AHPV screening using different triage strategies were different across different age cohorts. Thus, AHPV may be an appropriate primary screening method for 35–44 and 55–64 year old females, while AHPV-GT with reflex LBC triage may be more apt for 35–44 year old females.

## Introduction

Cervical cancer (CC) is the 4^th^ most frequent cancer and the 4^th^ most common cause of cancer-related death in females (Sung et al., 2021). High-risk human papillomavirus (hr-HPV) persistence is a necessary cause (Rijkaart et al., 2012). Using this etiology, primary HPV (pHPV) screening is used to detect cervical precancerous disease and cancer in the general population (Huh et al., 2015; Ronco et al., 2010; Ogilvie et al., 2018). Currently, the Food and Drug Administration (FDA) has approved four HPV assays, including one RNA-based and three DNA-based assays (Iftner et al., 2015). Previous investigations reported that HPV E6/E7 mRNA assay data (Aptima HPV, AHPV) were consistent with DNA assay data, showed similar sensitivity for cervical intraepithelial neoplasia (CIN)2+ or CIN3+, but showed slightly higher specificity than DNA assays (Ge et al., 2018; Zhang et al., 2020). It was also reported that AHPV assays were acceptable for primary CC screening using clinician-collected cervical samples at approximate 5 years intervals (Arbyn et al., 2022).

As most HPV infections spontaneously clear, HPV testing identifies many infections that do not progress to cervical precancerous disease or cancer, especially in young females (Ho et al., 1998). Therefore, primary HPV (pHPV) screening is advocated in females ≥ 30 years old (World Health Organization, 2021). Several investigations have also reported the pooled effectiveness of HPV testing in females aged ≥ 30–35 years old, as HPV prevalence in this group is lower than that in younger women (Bao et al., 2022). However, in China, HPV infections in females display two-peak patterns, one in 21–24-year olds and another in 55–64-year olds (Wang et al., 2022; Bao et al., 2021). Moreover, Chinese society is now aging, and older females have become the focus of increased screening (Xia et al., 2022). The second peak increases the number of females with transient HPV infections, which complicates triage strategy selection for this group. Critically, there is a dearth of prospective cohort studies with large sample sizes for age-specific AHPV performance, therefore, this requires exploration.

To address this knowledge gap, we examined the performance of age-specific primary AHPV screening versus cytology only, and evaluated the performance of different age-specific triage methods to identify optimal methods for Chinese females.

## Materials and methods

### Study population

Between April 2018 and December 2021, we recruited females from Liaoning Province (Shenyang City and Benxi County represented urban populations and Sujiatun District represented urban populations) and also Qingdao City (urban population). Inclusion criteria were (1) registered residents (resident for > 3 years) in the screening district; (2) 35–64 year old females; (3) no recorded severe organ dysfunction/mental illnesses; (4) no CC, hysterectomy, or pelvic radiation therapy history; and (5) voluntary participation and a demonstrable ability to complete questionnaires. As exclusion criteria, we excluded pregnant or lactating females, and those with limited samples for HPV testing/cytology.

Using reproductive stage, three groups were formed: 35–44-year olds (fertile), 45–54-year olds (peri-menopause), and 55–64-year olds (post-menopause). We received ethical approval from our local ethics committee (20180106) and all participants provided written informed consent.

### Liquid-based cytology (LBC)

Cytobrush-collected exfoliated cervical cells were added to PreservCyt collection medium (Hologic Inc., Marlborough, MA, USA) and used for LBC and AHPV assays (Hologic, San Diego, CA, USA). All specimens were initially analyzed using ThinPrep^®^ LBC (Hologic Inc.). LBC results were evaluated using the Bethesda System (2014) (Nayar and Wilbur, 2015).

### HPV testing and genotyping

Remaining exfoliated cells were blindly assayed using Aptima^®^ HPV assays, which are FDA-certified HPV E6/E7 mRNA assays that detect 14 high risk (hr)-HPV types (HPV16, 18, 31, 33, 35, 39, 45, 51, 52, 56, 58, 59, 66, and 68). All hr-HPV-positive samples were genotyped using the Aptima^®^ HPV16 18/45 genotype (GT) assay (AHPV-GT) (Gen Probe; Hologic, San Diego, CA, USA), which detects HPV16 and a subset of HPV18 and HPV45 cases (Wang et al., 2019). Analyses were performed according to manufacturer’s instructions.

### Colposcopy and biopsy

All colposcopies were performed by highly trained personnel. The referral criteria for colposcopy were as follows: (1) atypical squamous cells of undetermined significance (ASC-US) or a worse cytological diagnosis; (2) HPV-positive result; (3) if visible abnormalities were evident, colposcopy was immediately conducted regardless of screening outcomes. For any abnormal epithelium, colposcopy-guided biopsy was conducted. Biopsy results were categorized as follows: normal (no pathological alterations and cervicitis), low-grade squamous intraepithelial lesions (LSIL), and high-grade squamous intraepithelial lesions or worse (HSIL+). HSIL lesions were confirmed by p16 and Ki-67 immunohistochemistry. If the cytological result of participant was ASC-H, HSIL or AGC and no visible abnormality was found during colposcopic examination, random 3, 6, 9, and 12 o’clock position biopsies from the cervix and endocervical curettage were collected. Participants with a cytological ASC-US or LSIL diagnosis, no evidence of HPV 16/18 infection and a completely normal colposcopic impression did not undergo biopsy and were considered to have a histological status of “no HSIL”. Those with negative co-screening results also had a “no HSIL” histological status.

### Outcome measures

Histological HSIL+ confirmation was the clinical endpoint. LBC positivity at primary screening was defined as ASC-US or worse. AHPV positivity at primary screening was deemed positive for any hr-HPV infection. Two triage strategies were established for HPV-positive females: (1) AHPV-LBC triage: AHPV test-positive females underwent colposcopy if LBC generated an ASC-US result or worse, and (2) AHPV-GT with reflex LBC triage: AHPV-positive females were further HPV genotyped and underwent colposcopy if they were HPV16/18/45-positive, or positive for other hr-HPV genotypes with an LBC test result of ASC-US or worse.

### Data analyses

Using 95% confidence intervals (CI), age-specific positive screening rates, colposcopy referral rates, and HSIL+ detection rates were calculated. Absolute estimates and 95% CI for age-specific sensitivity, specificity, positive predictive values (PPV), and negative predictive values (NPV) were also calculated. McNemar tests were used to identify significant sensitivity and specificity differences. Area under the receiver operating characteristic curve (AUC) values were calculated using standard definitions and compared using Delong tests. We used Pearson’s chi-squared tests to compare PPVs and NPVs. Categorical variable differences between groups were examined using chi-squared tests. SPSS 22.0 and R software v.3.5.4 (R Foundation for Statistical Computing, Vienna, Austria) programs were used and P < 0.05. represented statistical significance.

## Results

### General cohort traits

**Figure 1:**
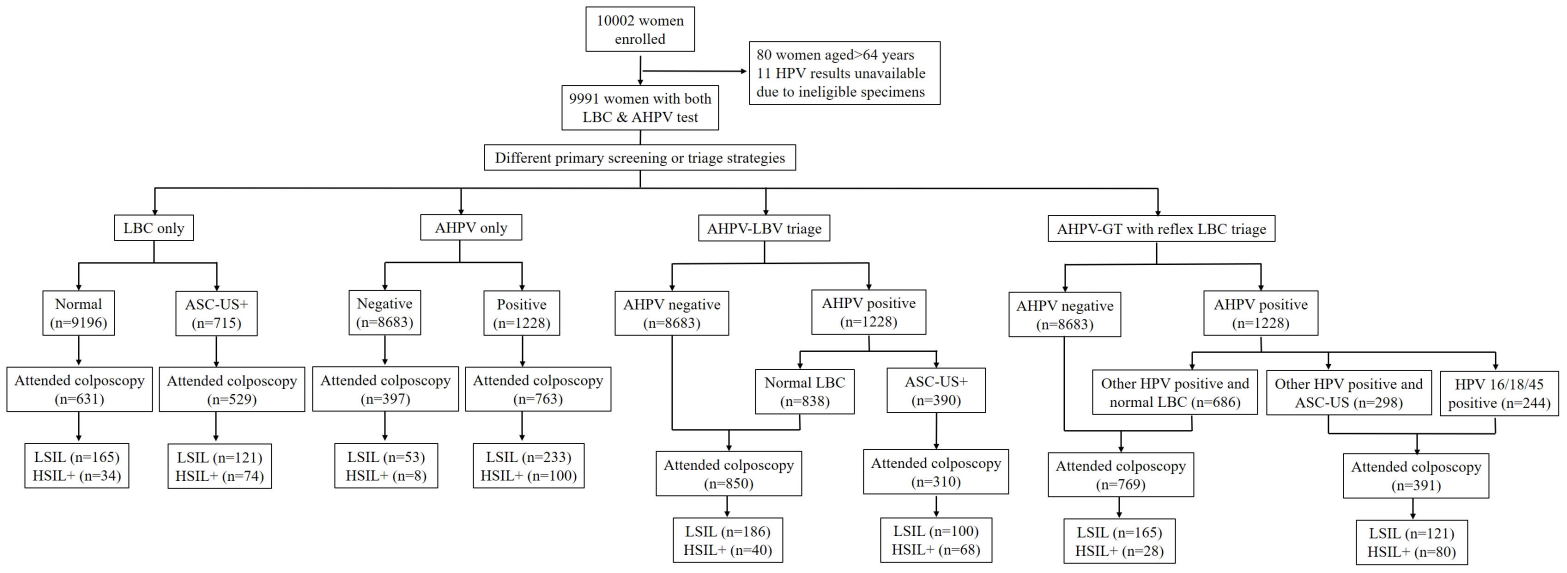
Flow diagram showing the study population.

We recruited 10,002 females in total, but excluded those for whom HPV results were unavailable (ineligible specimens) and those aged < 35 or ≥ 65 years old. Therefore, we processed data from 9911 females (**Figure 1**). Of these, 7891 (79.6%) were from Liaoning province and 2020 (20.4%) were from Qingdao City; 5915 (59.7%) came from an urban population and 3996 (40.3%) from a rural population. The median age = 49 years old (interquartile range = 44∼55). Population demographics analyses indicated that of the 9911 females, 5404 (54.5%) were peri-menopausal and 4507 (45.5%) were post-menopausal. Additionally, 9304 (93.9%) had no smoking history, 527 (5.3%) were smokers, and 80 (0.8%) had previously quit smoking. Moreover, 5539 (55.9%) had had two or more pregnancies, while 1021 (10.3%) had two or more deliveries. All participants were unvaccinated. Overall, 7708 (77.8%) had never previously had CC screening. Total screening-positive tests for all ages, for which colposcopy referral was required, varied from 390 (3.9%) to 1228 (12.4%) for different screening strategies. Colposcopy was performed on 62.1%–79.5% of positive females using different screening methods, and 96.8%–97.4% of females had adequate negative colposcopy findings or an adequate biopsy specimen.

Of the 9911 participants, 715 (7.2%) had abnormal cytology (ASC-US or worse) and 1228 (12.4%) had HPV-positive results (**Figure 1**). In total, 108 (1.1%) females with HSIL+ were identified, of which 5 (0.5c) had confirmed CC. The age-specific distribution of female clinical characteristics was shown (**Table 1**). Age-specific abnormal cytology rates were 6.1%–8.0%, which were highest in 45–54-year olds and lowest in 35–44-year olds (P = 0.002). Moreover, low-grade abnormal cytology rates were significantly higher in 45–54-year olds (P < 0.05). HPV prevalence varied greatly across cohorts; rates in 55–64-year olds (14.1%) were higher than those in 35–44-year olds and 45–54-year olds (12.2%, P = 0.048 and 11.6%, P = 0.002, respectively). Histological HSIL+ detection rates did not significantly differ across cohorts. LSIL detection rates in 55–64-year olds were significantly higher than those in 35–44-year olds (3.5% vs. 2.5%, P = 0.028).

**Table 1:** Age-specific distribution of clinical characteristics of women screened.

Screening test	35-44-year olds N=2603 (%)	45-54-year olds N=4706 (%)	55-64-year olds N=2602 (%)	Total N=9911 (%)	χ^2^	*p*
LBC abnormal	158 (6.1)	378 (8.0)	179 (6.9)	715 (7.2)	9.497	0.002^*^
					1.408	0.235^**^
					3.164	0.075^***^
Low-grade	128 (4.9)	332 (7.1)	152 (5.8)	612 (6.2)	12.983	<0.001^*^
					2.184	0.139^**^
					3.988	0.046^***^
High-grade	30 (1.2)	46 (1.0)	27 (1.0)	103 (1.0)	0.499	0.480^*^
					0.158	0.691^**^
					0.061	0.804^***^
AHPV Positive	318 (12.2)	544 (11.6)	366 (14.1)	1228 (12.4)	0.695	0.404^*^
					3.899	0.048^**^
					9.655	0.002^***^
Histological diagnosis						
LSIL	65 (2.5)	129 (2.7)	92 (3.5)	286 (2.9)	0.386	0.534^*^
					4.798	0.028^**^
					3.607	0.058^***^
HSIL+	29 (1.1)	43 (0.9)	36 (1.4)	108 (1.1)	0.690	0.406^*^
					0.766	0.381^**^
					3.459	0.063^***^

LBC, liquid-based cytology; AHPV, the Aptima human papillomavirus assay; LSIL, low-grade squamous intraepithelial lesion; HSIL, high-grade squamous intraepithelial lesion.

^*^The comparison between 35-44 years and 45-54 years. ^**^The comparison between 35-44 years and 55-64 years. ^***^The comparison between 45-54 years and 55-64 years.

### The age-specific performance of different primary screening assays and triage methods to detect HSIL+

The performance of AHPV varied by age. AHPV sensitivity to detect HSIL+ in 55–64-year olds (97.2 [95% CI: 91.7–100]) was higher than that in 45–54-year olds (86.0 [95% CI: 74.4–95.3]). The PPV of AHPV in 35–44-year olds (17.7 [95% CI: 12.3–24.8]) was higher than that in 45–54-year olds (10.2 [95% CI: 7.3–13.9]).

In 35–44-year olds (**Table 2**), AHPV sensitivity was 96.6 (95% CI: 89.7–100), which was significantly higher than LBC sensitivity (65.5 [95% CI: 48.3–82.8], P < 0.001). No significant differences were observed between PPVs for AHPV and LBC (17.7 [95% CI: 12.3–24.8] vs. 18.3 [95% CI: 11.6–27.3], P = 0.910). The AUC value for the AHPV assay was significantly higher than the LBC test value (0.956 [95% CI: 0.921–0.990] vs. 0.810 [95% CI: 0.722–0.898], P < 0.001). In 35–44-year olds, when compared with LBC, the detection rates for AHPV-GT with reflex LBC triage for HSIL+ increased 31.5% (9.6‰ vs. 7.3‰), and colposcopy referral rates decreased 16.4% (5.1% vs. 6.1%). The AUC value for AHPV-GT with reflex LBC triage (0.918 [95% CI: 0.855–0.982]) was significantly higher than the LBC value (0.810 [95% CI: 0.722–0.898], P = 0.0046). So, the performance of AHPV and AHPV-GT with reflex LBC triage was better than that of LBC in the detection of HSIL+ in 35–44-year olds.

**Table 2:** Comparison of performance of different primary screening tests and different triage strategies.

Screening tests	No. of HSIL+ detected	HSIL+ detection rate (‰)	The rate of referred to colposcopy(%)	Sensitivity(95%*CI*)	Specificity(95%*CI*)	PPV(95%*CI*)	NPV(95%*CI*)	AUC(95%*CI*)	*P*
35-44-year olds									
LBC	19	7.3	6.1	65.5 (48.3, 82.8)	96.4 (95.7, 97.2)	18.3 (11.6, 27.3)	99.6 (99.2, 99.8)	0.810 (0.722, 0.898)	
AHPV	28	10.8	12.2	96.6 (89.7, 100.0)	94.6 (93.7, 95.4)	17.7 (12.3, 24.8)	100.0 (99.7, 100.0)	0.956 (0.921, 0.990)	<0.001^*^
AHPV-LBC	19	7.3	3.6	65.5 (48.3, 82.8)	98.1 (97.5, 98.6)	29.2 (18.9, 42.0)	99.6 (99.2, 99.8)	0.818 (0.730, 0.906)	NA^**^
AHPV-genotyping with reflex LBC	25	9.6	5.1	86.2 (67.4, 95.5)	97.5 (96.9, 98.1)	29.4 (20.3, 40.4)	99.8 (99.5, 99.9)	0.918 (0.855, 0.982)	0.0046^#^
45–54-year olds									
LBC	31	6.6	8.0	72.1 (58.1, 83.7)	94.2 (93.5, 94.9)	10.8 (7.6, 15.1)	99.7 (99.5, 99.8)	0.832 (0.764, 0.900)	
AHPV	37	7.9	11.6	86.0 (74.4, 95.3)	92.6 (91.9, 93.4)	10.2 (7.3, 13.9)	99.9 (99.7, 100.0)	0.893 (0.841, 0.946)	0.178^*^
AHPV-LBC	26	5.5	4.2	60.5 (46.5, 74.4)	97.0 (96.5, 97.5)	16.3 (11.1, 23.1)	99.6 (99.4, 99.8)	0.787 (0.713, 0.861)	0.073^**^
AHPV-genotyping with reflex LBC	29	6.2	5.4	67.4 (53.5, 81.4)	96.3 (95.7, 96.9)	15.0 (10.5, 21.0)	99.7 (99.4, 99.8)	0.819 (0.748, 0.890)	0.698^#^
55-64-year olds									
LBC	24	9.2	6.9	66.7 (50.0, 80.6)	95.8 (95.0, 96.7)	19.4 (13.0, 27.6)	99.5 (99.1, 99.7)	0.812 (0.734, 0.891)	
AHPV	35	13.5	14.1	97.2 (91.7, 100.0)	92.3 (91.3, 93.4)	16.0 (11.5, 21.7)	100.0 (99.7, 100.0)	0.948 (0.920, 0.975)	0.002^*^
AHPV-LBC	23	8.8	3.9	63.9 (50.0, 80.6)	97.8 (97.2, 98.4)	30.7 (20.8, 42.5)	99.4 (99.0, 99.7)	0.809 (0.729, 0.888)	0.783^**^
AHPV-genotyping with reflex LBC	26	10.0	6.0	72.2 (58.3, 86.1)	96.9 (96.2, 97.6)	25.7 (17.8, 35.6)	99.6 (99.2, 99.8)	0.845 (0.771, 0.920)	0.236^#^

AHPV-LBC: AHPV test positive cases were referral if LBC test with ASCUS or worse. AHPV-genotyping with reflex LBC, AHPV positive cases were further tested by HPV genotyping, and referred to colposcopy if HPV16, 18/45 positive, or if other HR-HPV genotypes positive with LBC test ASCUS or worse.

^*^Comparison of AUC between LBC and AHPV. ^**^Comparison of AUC between LBC and AHPV-LBC.

^#^Comparison of AUC between LBC and AHPV-genotyping with reflex LBC.

HSIL high-grade squamous intraepithelial lesion; PPV positive predictive value; NPV negative predictive value; AUC area under the receiver operating characteristic curve.

NA, not applicable.

In 45–54-year olds (**Table 2**), although AHPV sensitivity was 86.0 (95% CI: 74.4–95.3), which was higher than the LBC value (72.1, 95% CI: 58.1–83.7), the difference was not significant (P = 0.210). Also, while the AHPV AUC value (0.893 [95% CI: 0.841– 0.946]) was slightly higher than the LBC value (0.832 [95% CI: 0.764–0.900]), the difference was not significant (P = 0.178). The HSIL+ detection rate for AHPV-GT with reflex LBC triage was lower than the LBC rate (6.2‰ vs. 6.6‰).

In 55–64-year olds (**Table 2**), HSIL+ sensitivity using AHPV was higher (97.2 [95% CI: 91.7–100.0]) when compared to LBC sensitivity (66.7 [95% CI: 50.0–80.6], P = 0.003). However, AHPV specificity was lower when compared to LBC specificity (92.3 [95% CI: 91.3–93.4] vs. 95.8 [95% CI: 95.0–96.7], P < 0.001), while PPV differences between AHPV and LBC assays were not statistically significant (16.0 [95% CI: 11.5–21.7] vs. 19.4 [95% CI: 13.0–27.6], P = 0.426). AHPV AUC values were significantly greater when compared to LBC test values (0.948 [95% CI: 0.920–0.975] vs. 0.812 [95% CI: 0.734–0.891], P = 0.002). In 55–64 year old females, when compared with LBC, detection rate of AHPV-GT with reflex LBC triage for HSIL+ increased 8.7% (10.0‰ vs. 9.2‰) and colposcopy referral rate decreased 13.0% (6.0% vs. 6.9%). However, AUC values were not significantly different between AHPV-GT with reflex LBC triage and LBC (0.845 [95% CI: 0.771–0.920] vs. 0.812 [95% CI: 0.734–0.891], P = 0.236). In this age group, the performance of AHPV-GT with reflex LBC triage had no apparent competitive superiority to that of LBC in the detection of HSIL+.

## Discussion

In this real-world, large community population, we investigated the age-specific performance of AHPV assays versus cytology in detecting HSIL+ in females in the 35–64 year old age range. While HPV testing is proposed for females aged ≥ 30 years, challenges remain in terms of determining optimal triage methods for HPV-positive females (World Health Organization, 2021; Melnikow et al., 2018). We identified better AHPV performance when compared with LBC in 35–44-year and 55–64-year olds. Moreover, AHPV-GT with reflex LBC triage was suitable for 35–44-year olds. However, this approach was not entirely effective for 55–64-year olds, and in 45–54-year olds, it was inferior to LBC in terms of detecting HSIL+.

We observed that hr-HPV prevalence was highest in 55–64-year olds (14.1%), followed by 35–44-year olds (12.2%), and 45–54-year olds (11.6%). Consistent with these observations, recent Chinese studies have reported a second HPV infection peak in females aged 50–60 (Zhang and Zhang, 2023) or 55–64 years old (Bao et al., 2021).

When compared to LBC, more HSIL+ cases were detected by the AHPV method irrespective of age in the present study. However, AHPV sensitivity was significantly better for females aged 35–44 years old (P < 0.001) and 55–64 years old (P = 0.003), whereas AHPV and LBC showed comparable sensitivity in 45–54-year olds (P = 0.210). Some previous investigations have reported the age-specific performance of HPV testing using HPV DNA methods in primary CC screening (Leinonen et al., 2009; Schiffman et al., 2000; Labani and Asthana, 2016). For instance, a Costa Rican investigation reported higher HPV testing sensitivity in their ≥ 41-year old group (93.2%) when compared to the 31–40-year old group (80.8%) (Schiffman et al., 2000). An Indian investigation reported the highest CHPV testing sensitivity in a ≥ 50-year old group (Labani and Asthana, 2016). In the present study, AHPV sensitivity was the highest (97.2%) in 55–64-year olds and the lowest (86.0%) in 45–54-year olds. We also observed that hr-HPV prevalence was the highest (14.1%) in 55–64-year olds and the lowest (11.6%) in 45–54-year olds. It follows that associations between age and HPV testing sensitivity may differ across studies, owing to cohort effects and variations in HPV population prevalence which depends on age-specific societal sexual practices and geography (Geisinger et al., 2021). Moreover, according to these three investigations, we found that primary HPV screening by both HPV DNA and AHPV test had good sensitivity in the older age group. In contrast, other investigations identified uniformly higher HPV sensitivity, which was unaffected by age (Cuzick et al., 2006; Nygård et al., 2022; Zhao et al., 2010). In our investigation, AHPV specificity decreased with increasing age. Likewise, it was previously reported that HPV test specificity varied with age (P < 0.0001) and was highest in females < 35 years old (Zhao et al., 2010). However, it was reported elsewhere that HPV testing specificity increased with increasing age (Cuzick et al., 2006). In our investigation, the potential biological or epidemiological factors might contribute to these age-specific differences in AHPV screening. First, females aged 45–54 year old with the lowest hr-HPV prevalence are in perimenopause, with fluctuating sex hormone levels and reduced frequency of sexual behavior. Secondly, lower HPV testing specificity in older females was due to a higher prevalence of HPV infection. Females aged 55–64 years old are often more susceptible to HPV infection because their immune systems are less potent, and the epithelium of the cervix and vagina reportedly becomes thinner and may undergo microdamage (Andersen et al., 2020). However, our LSIL detection rates were higher in 55–64-year olds females.

LBC had the highest sensitivity in 45–54-year olds, followed by 55–64-year olds. Consistent with a previous investigation, cytology demonstrated substantially better sensitivity in detecting HSIL+ in females > 50 years old when compared with younger females (Cuzick et al., 2006). This was possibly due to the age-specific prevalence of abnormal cytology (Al Zaabi et al., 2015). Previous study reported that the proportion of abnormal cytology decreased with increasing age, however, in older women, cytological abnormalities are largely high-grade lesions (Campaner and Fernandes, 2023). We observed that the prevalence of abnormal cytology (8.0%) was highest in 45–54-year olds. In perimenopausal and postmenopausal females, typical cervical intraepithelial lesion cells are not easily misdiagnosed by cytologists, while cell atrophy caused by low estrogen levels are similarly detected by cytologists.

When HPV testing was recommended as a primary screening test (World Health Organization, 2021), optimal triage strategies should have been reconsidered. In our investigation, we observed that the effectiveness of two triage strategies for HPV-positive females differed depending on age. When compared with LBC, the AHPV-LBC triage approach showed similar HSIL+ detection rates in 35–44 year olds, and lower HSIL+ detection rates in 45–54 and 55–64 year olds. The AHPV-GT with reflex LBC triage approach detected more HSIL+ cases when compared with LBC in 35–44 and 55–64 year olds, but not in 45–54 year olds. Accordingly, referral rates for colposcopy in the AHPV-GT with reflex LBC triage group increased with increasing age (5.1% in 35–44 year olds, 5.4% in 45–54 year olds, and 6.0% in 55–64 year olds). Also, AUC values did not differ significantly between AHPV-GT with reflex LBC triage and LBC methods in 55–64 year olds. Bao et al. (Bao et al., 2022) reported that in their HPV testing/genotyping with reflex cytology triage cohort, more HSIL+ cases were recorded when compared with their cytology only group in 35–54 year olds, but not in 55–64 year olds. A possible reason was that a large proportion of HSIL+ cases in postmenopausal females may have resulted from infections by HPV non-16/18 types (Gyllensten et al., 2012). Therefore, both approaches are limited for perimenopausal and postmenopausal HPV-positive triage in females, thus alternative triage methods are required.

In terms of study strength, ours was a truly representative investigation of HPV testing in Chinese urban and rural communities, rather than in hospital settings. However, we also identified some limitations. Firstly, colposcopy referral compliance must be improved for HPV-positive but cytology-negative females. Secondly, we only considered three age groups, therefore females aged 25–34 years old must be considered in future research. Finally, longitudinal screening investigations are required to improve age-specific triage methods in pHPV screening.

In conclusion, primary AHPV screening using different triage methods differed across different age groups. AHPV may be an appropriate primary screening method for females aged 35–44 and 55–64 years old. AHPV-GT with reflex LBC triage may be used for females aged 35–44 years. Finally, in this era of pHPV testing, different triage methods should be reconsidered for older HPV-positive females.

## Data Availability

The raw data supporting the conclusions of this article will be made available by the authors, without undue reservation.
